# Supplementary material for: Synthesis, Optical, and Geometrical Approaches of New Natural Fatty Acids’ Esters/Schiff Base Liquid Crystals
Source: Molecules. 2019 Nov 25;24(23):4293. doi: 10.3390/molecules24234293 (PMC6930458; doi:10.3390/molecules24234293)
Supplement: Supplementary file 1 [file molecules-24-04293-s001.pdf]

Supplementary Data

Synthesis, Optical, and Geometrical Approaches of New Natural Fatty Acids' Esters/Schiff Base Liquid Crystals

Rua Alnoman [1], Fares khalid Al-nazawi[2], Hoda A. Ahmed [1,3,*] and Mohamed Hagar [1,4,*]

[1] College of Sciences, Chemistry Department, Taibah University, Yanbu 30799, Saudi Arabia. rua-b-n@live.co.uk
[2] Ibn al-Nafis Secondary School, Yanbu Industrial, Yanbu 46455, Saudi Arabia. Faris-kh16@hotmail.com
[3] Faculty of Science, Department of Chemistry, Cairo University, Cairo 12613, Egypt
[4] Faculty of Science, Chemistry Department, Alexandria University, Alexandria 21321, Egypt
* Correspondence: ahoda@sci.cu.edu.eg (H.A.A.); mohamedhaggar@gmail.com (M.H.); Tel.: +966-545527958 (M.H.); +966-0542015471 (H.A.A.)

Materials

4-Hydroxybenzaldehyde, 4-hexyloxyanilene palmitic acid, oleic acid, and linoleic acid were obtained from Sigma Aldrich (Germany). *N,N'*- dicyclohexylcarbodiimide (DCC), 4-dimethylaminopyridine (DMAP),dichloromethane ethanol and methanol were obtained from Aldrich (Wisconsin, USA).

Characterization of synthesized compounds

The purity of the prepared samples was checked with TLC sheets coated with silica gel (E Merck)

Infrared spectra were measured using Perkin-Elmer B25 (Perkin-Elmer, Inc., Shelton, CT USA) spectrophotometer. ^{1}HNMR spectra were recorded using a Varian EM 350L 300 MHz spectrometer (Oxford, UK) using tetramethylsilane as internal standard and $CDCl_3$ as solvent; the chemical shift values recorded as δ (ppm units).

Elemental analyses for final products were carried out on Thermo Scientific Flash 2000 CHS/O Elemental Analyzer, Milan, Italy.

The spectrophotometer technique (UV-1800 SHIMADZU, Japan) of a wavelength ranging from 200–800 nm with normal incidence of light at 27 ^{o}C was used for all compounds. The solutions of the compounds were prepared in concentrations IA (2.1 $*10^{-5}$ mole/L), IIA (1.3 $*10^{-5}$ mole/L) and IIIA (2.1 $*10^{-5}$ mole/L).

Calorimetric measurements were carried out using a TA Instruments Co. Q20 Differential Scanning Calorimeter (DSC; USA). The DSC was calibrated using the melting temperature and enthalpy of indium and lead. DSC investigation was carried out for small samples (2–3 mg) placed in aluminum pans. All measurements were achieved at a heating rate of 10°C/min in inert atmosphere of nitrogen gas (30 ml/min) and all transition recorded from the second heating scan.

Transition temperatures were checked and types of mesophases identified, for all compounds prepared and their binary mixtures, with a standard polarized light microscope (PLM, Wild, Germany) attached with Mettler FP82HT hot stage.

Computational Method

Gaussian 09 software was used for DFT calculations for the studied compounds [1]. DFT/B3LYP methods using 6-31G (d,p) basis set was selected for the calculations. The geometries

were optimized by minimizing the energies with respect to all geometrical parameters without imposing any molecular symmetry constraints. The structures of the optimized geometries had been drawn with Gauss View [2]. Also, calculations frequencies were carried out by the same level of theory. The frequency calculations showed that all structures were stationary points in the geometry optimization method with none imaginary frequencies.

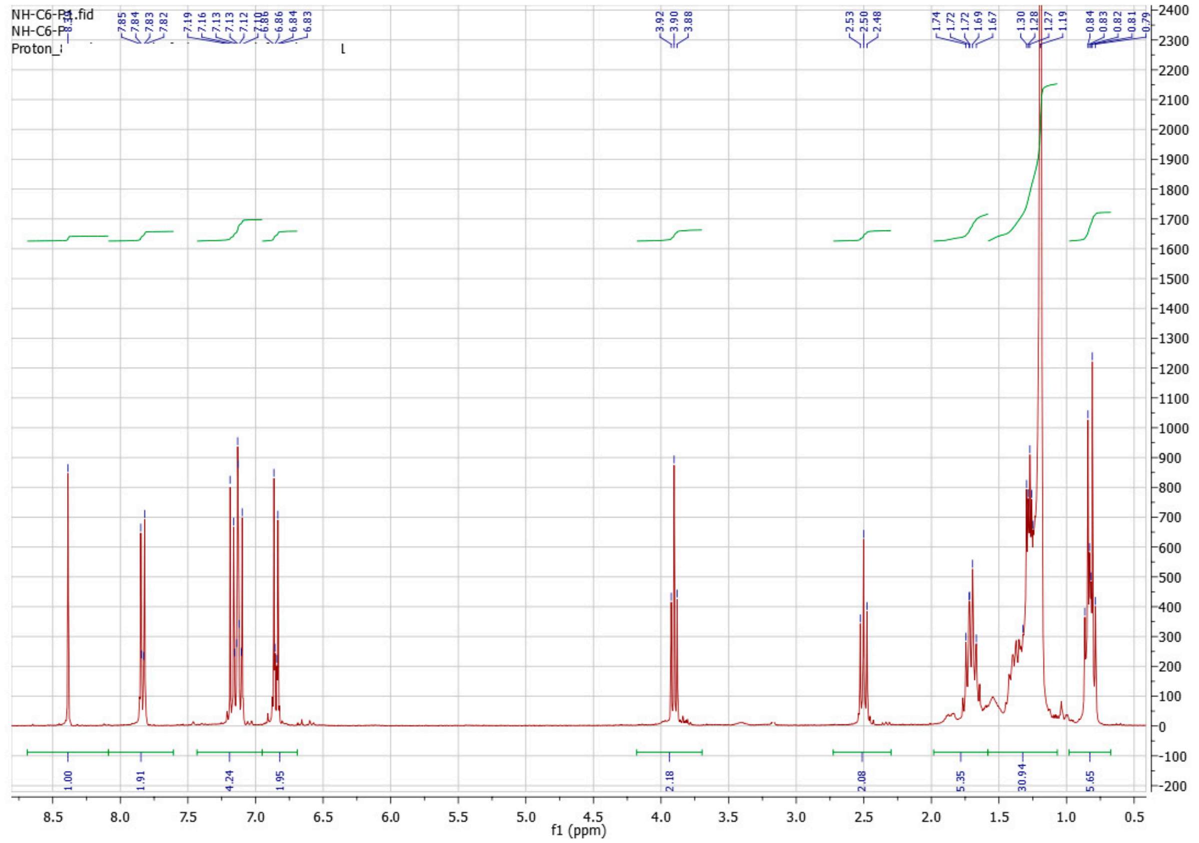

Figure S1: H^1 NMR of *4-((E)-9-cis-[4-(Hexyloxy)phenylimino)methyl]phenyl hexadec-9-enoate* **IA**

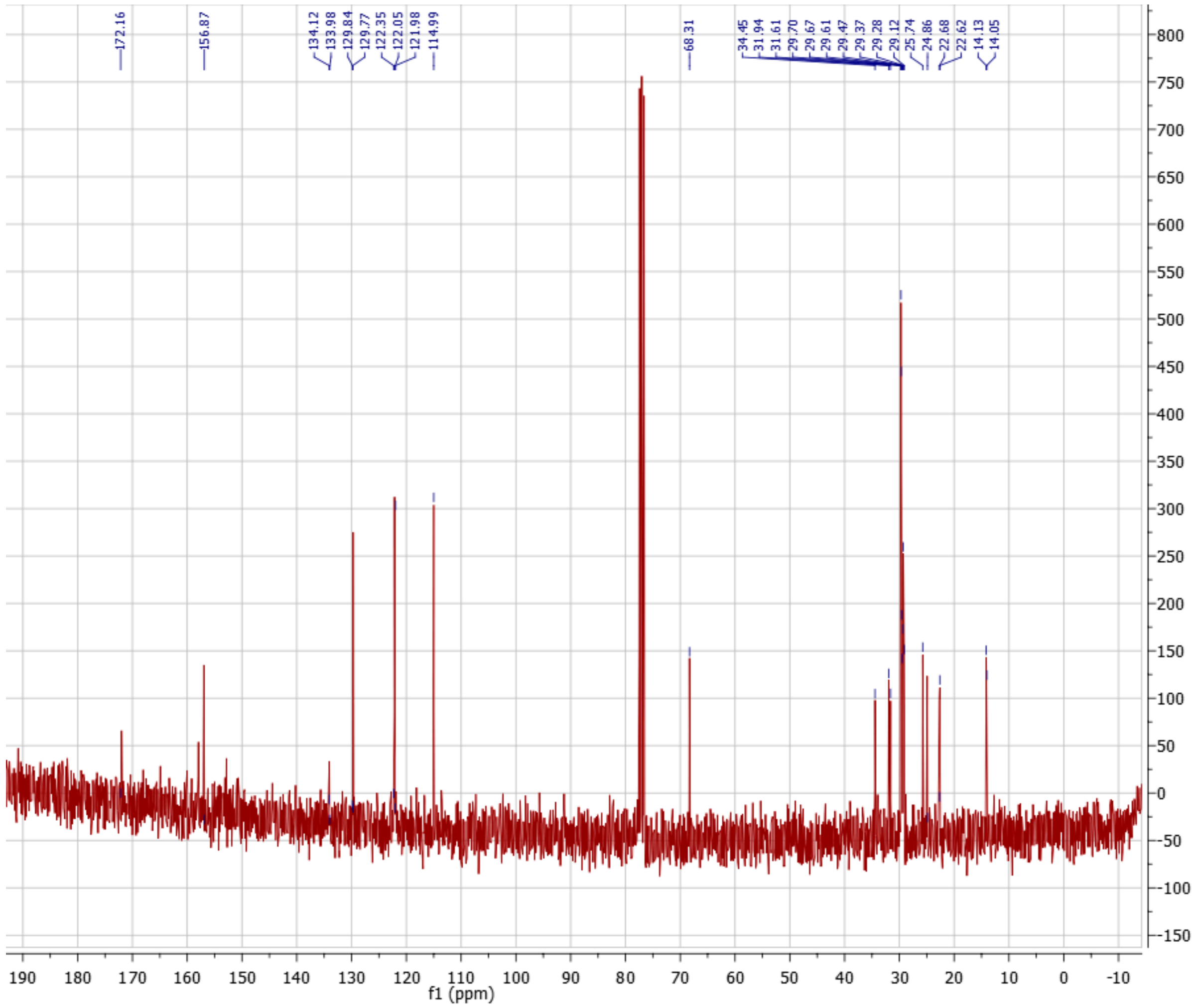

Figure S2: ^{13}C NMR of *4-((E)-9-cis-[4-(Hexyloxy)phenylimino)methyl]phenyl hexadec-9-enoate* **IA**

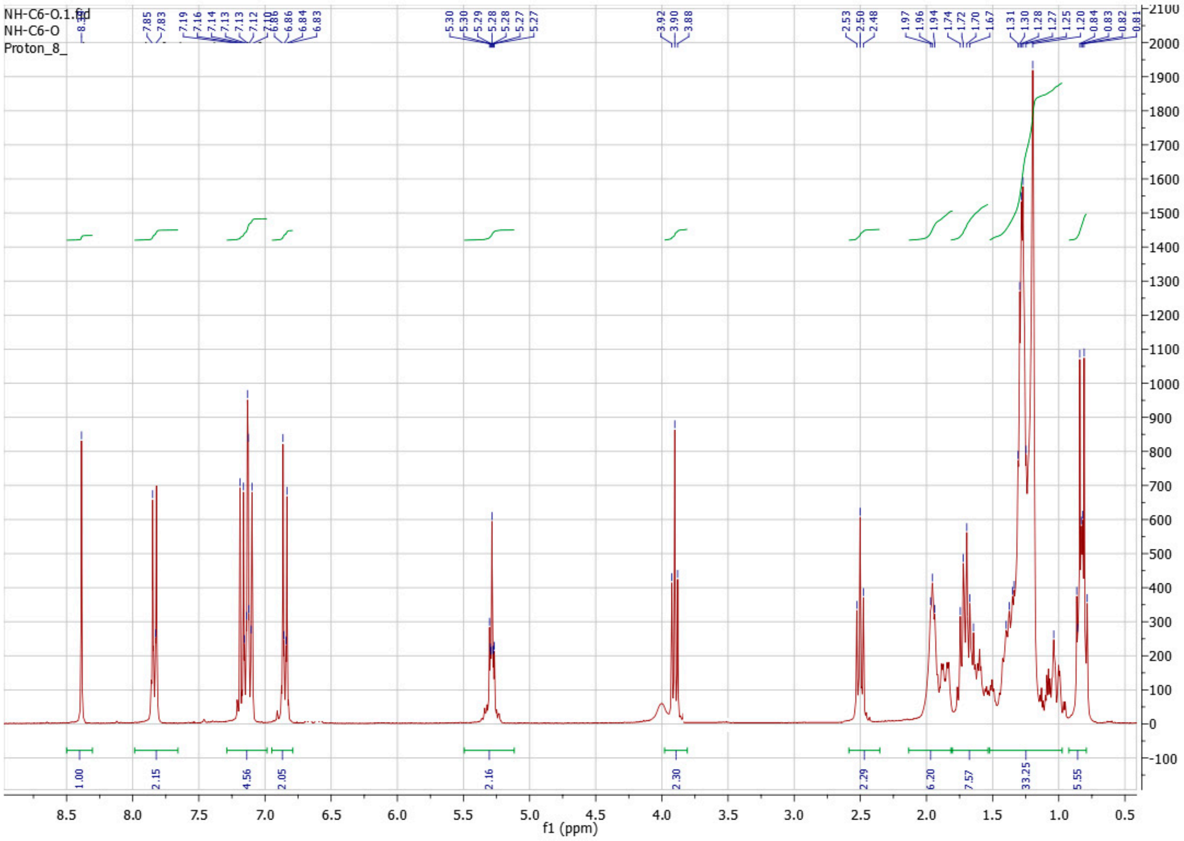

Figure S3: H[1] NMR of *4-((E)-9-cis-[4-(Hexyloxy)phenylimino)methyl]phenyl octadec-9-enoate* **IIA**

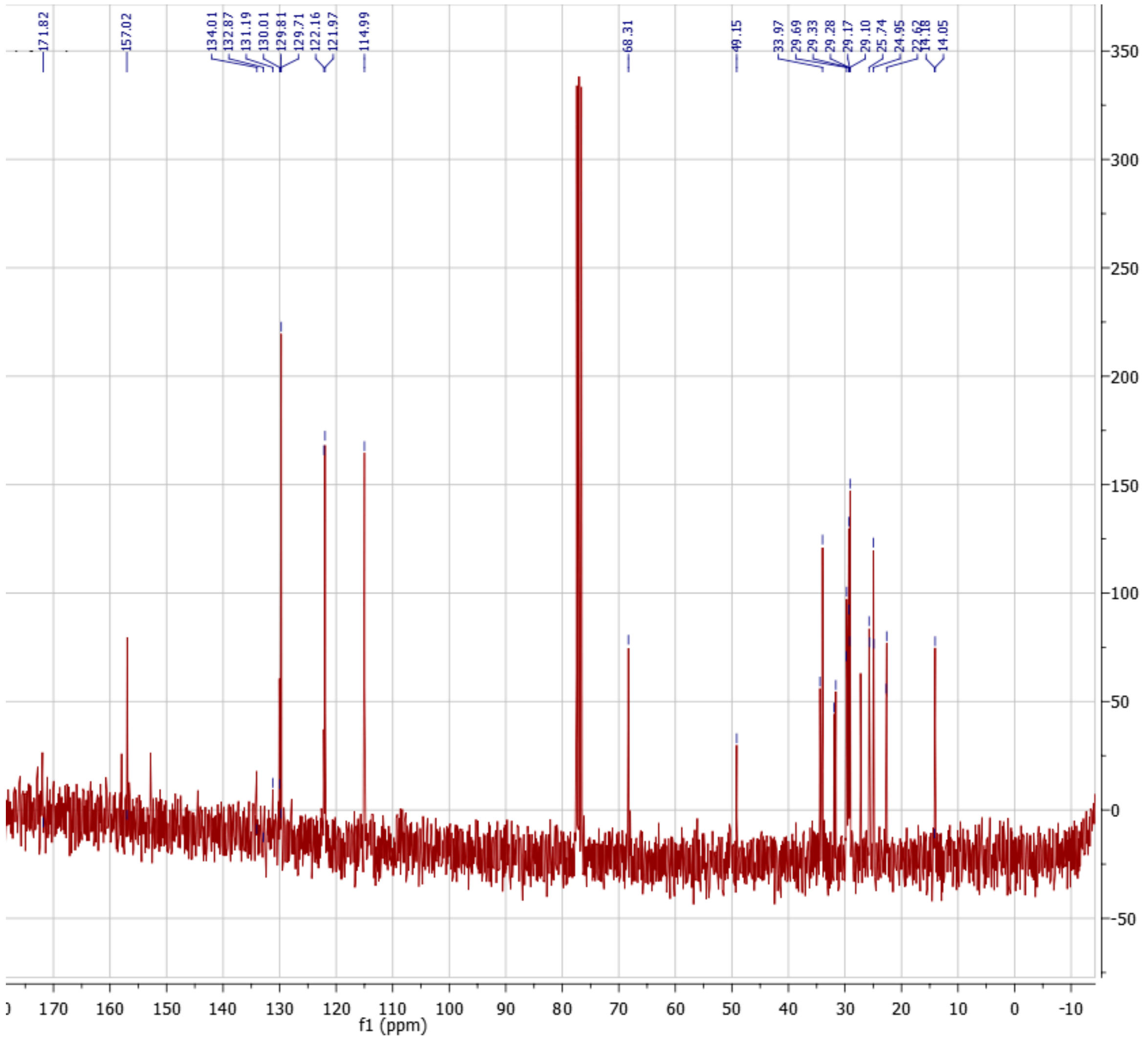

Figure S4: ^{13}C NMR of *4-((E)-9-cis-[4-(Hexyloxy)phenylimino)methyl]phenyl octadec-9-enoate* **IIA**

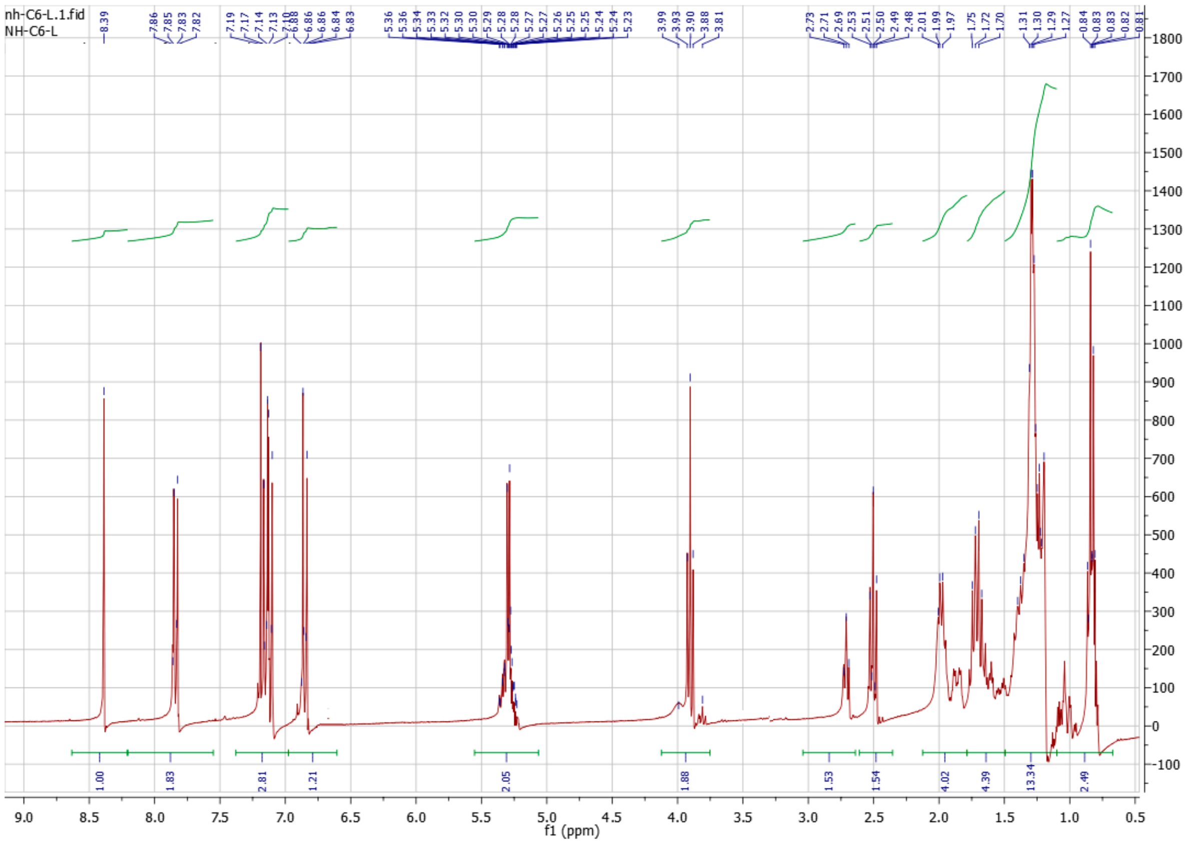

Figure S5: H¹ NMR of *4-((E)-9, 12-cis-[4-(Hexyloxy)phenylimino)methyl]phenyloctadeca-9,12-dienoate* **IIIA**

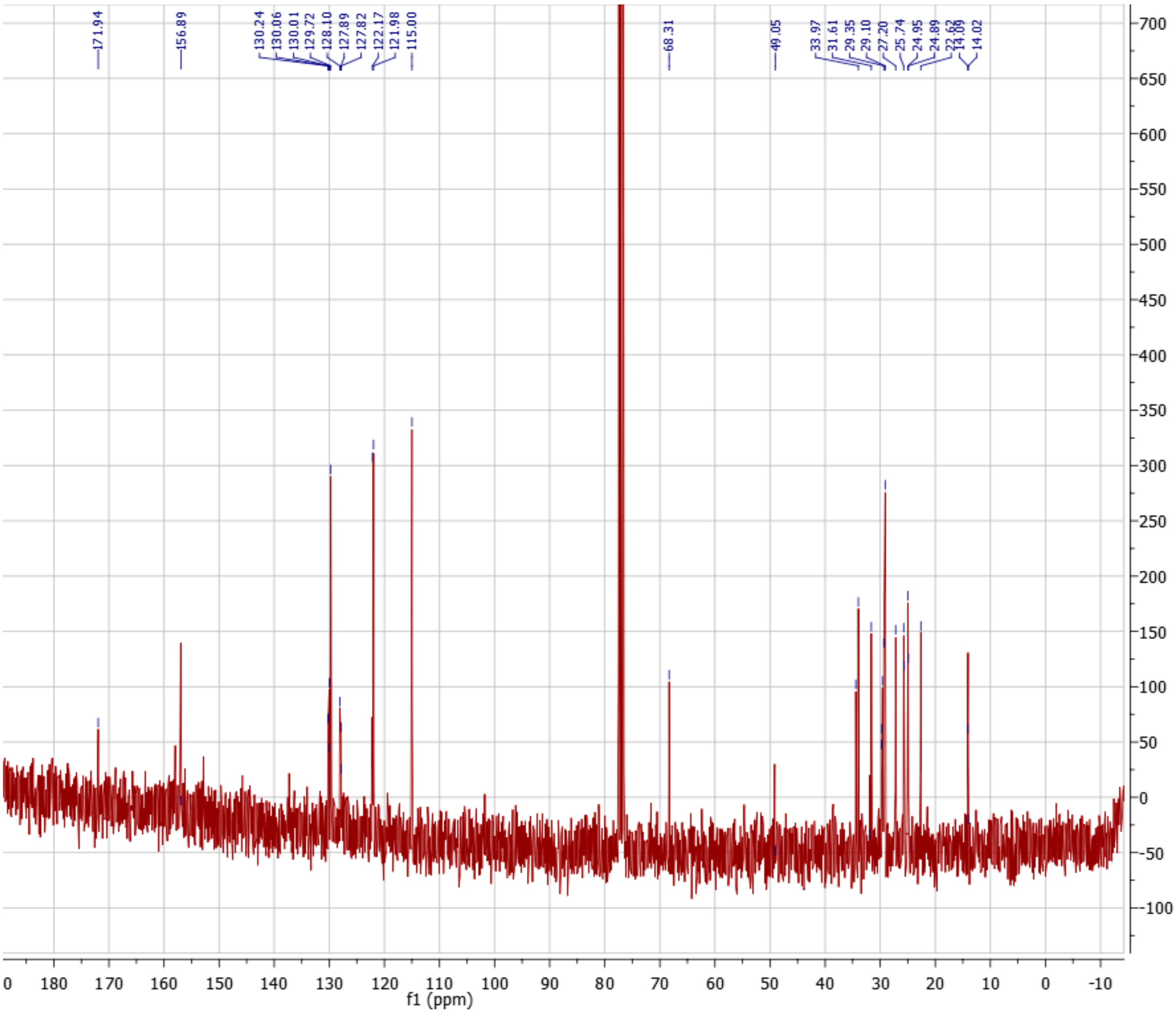

Figure S6: ^{13}C NMR of *4-((E)-9, 12-cis-[4-(Hexyloxy)phenylimino)methyl]phenyloctadeca-9,12-dienoate* **IIIA**

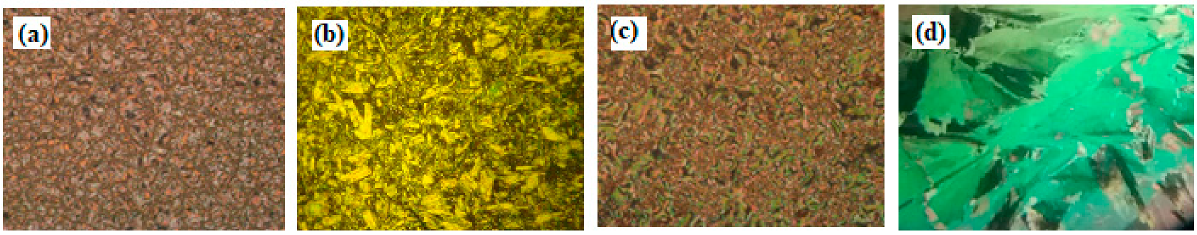

Figure S7**:** Liquid crystalline textures upon heating under POM of (a) SmC phase for compound **I** at 95.0 °C; (b) SmC phase for compound **II** at 69.0 °C; (c) SmC phase for compound **III** at 68.0 °C; and (d) SmA phase for compound **III** at 77.0 °C.

References

1. Frisch, M.; Trucks, G.; Schlegel, H. B.; Scuseria, G.; Robb, M.; Cheeseman, J.; Scalmani, G.; Barone, V.; Mennucci, B.; Petersson, G., Gaussian 09, revision a. 02, gaussian. *Inc., Wallingford, CT* **2009,** 200.
2. Dennington, R.; Keith, T.; Millam, J., GaussView, version 5. **2009**.
